# Cytotoxicity and anti-microbial analysis of silver and graphene oxide bio nanoparticles

**DOI:** 10.6026/97320630016831

**Published:** 2020-11-30

**Authors:** Iffat Nasim, S Rajesh Kumar, V Vishnupriya, Zohra Jabin

**Affiliations:** 1 Department of Conservative Dentistry and Endodontics, Saveetha Dental College and Hospitals, Saveetha Institute of Medical and Technical Sciences, Saveetha University, India; 2 Department of Pharmacology, Saveetha Dental College and Hospitals, Saveetha Institute of Medical and Technical Sciences, Saveetha University, Chennai - 600 077, India; 3 Department of Biochemistry, Saveetha Dental College and Hospitals, Saveetha Institute of Medical and Technical Sciences, Saveetha University, Chennai - 600 077, India; 4 Divya Jyoti College of Dental Sciences, Modinagar, Uttar Pradesh, India

## Abstract

It is of interest to document the cytotoxicity and anti microbial analysis of silver and graphene oxide nanoparticles. The plant extracts from Andrographis paniculata and Ocimum sanctum Linn were used as reducing agent. The nanoparticles were characterized using UV-visible spectroscopy, FT-IR, XRD and TEM. The antimicrobial activity was completed for oral pathogens. Brine Shrimp Lethality assay was conducted for cytotoxicity. Thus, we show that silver and graphene oxide bio based nanoparticles have antimicrobial activity with minimum cytotoxic effects.

## Background

Nanoparticles finds application in Biomedicine [1]. They are often produced using physical and chemical methods [2]. These molecules are also produced using biological methods using plants, fungi and bacteria [3]. Silver nanoparticles have attracted attention as they have favorable electrical conductivity, chemical stability and antibacterial activity [4]. Graphene is a carbon based flat monolayer arranged in a two dimensional hexagonal structure with distinguished mechanical, electrochemical and physical properties [5]. Andrographis paniculata is known for its potent anti-inflammatory formulations [6]. Ocimum sanctum Linn. is known for the treatment of upper respiratory tract infections, bowel infections and renal calculi [7]. We synthesised Silver and graphene oxide nanoparticles using leaf extract of Andrographis paniculata and Ocimum sanctum Linn. Therefore, it is of interest to document the cytotoxicity and anti microbial analysis of silver and graphene oxide nanoparticles.

## Materials and Methods:

### Preparation of plant extract:

Andrographis paniculata and Ocimum sanctum Linn leaves were collected freshly and dried in shade for three days. After that they were powdered coarsely.0.5g of Andrographis paniculata and 0.5g of Ocimum sanctum Linn leaves powder was weighed and dissolved in 100ml of distilled water and mixed well. After that it was boiled for 5 minutes at 60-80°C using a heating mantle. The boiled extract was filtered through Whatman No.1 filter paper, and the supernatant was used.

### Synthesis of Silver nanoparticles:

10 ml of pure plant extract was added into the 90 ml of 1 mM of silver nitrate solution and mixed well. Then the solution was kept in an orbital shaker for further mixing. The visual changes were observed and the colour of the prepared solution slowly turned to dark brown. This indicated silver nanoparticle formation. The prepared solution was then subjected to UV Vis Spectrophotometer analysis to confirm the formation of nanoparticles. Then this solution was centrifuged at 8000 rpm for 10 minutes. The solution was then filtered using Whatman No.1 filter paper.

### Synthesis of Graphene oxide nanoparticles:

0.6g of graphite nano powder (Sisco Research Laboratories, Maharashtra, India.) and 0.2 g of sodium hydroxide (MERCK, Mumbai, India.) was dissolved in 50 ml of distilled water; to this 50 mL of plant extract was added and mixed well. Then the solution was kept in an orbital shaker and magnetic stirrer with hot plate for further mixing. The color change was noted and the nanoparticles formation was monitored. Then this solution was centrifuged at 8000 rpm for 10 minutes. The solution was then filtered using Whatman No.1 filter paper.

### Characterization of newly synthesized nanoparticles:

UV Visible Spectrophotometer analysis, TEM Analysis, X-ray Diffraction and FTIR analysis was done to confirm the synthesis of nanoparticles.

### Assessment of Cytotoxicity [Brine shrimp lethality assay]:

The artemia tank was filled with 6 L of distilled water; to that 50 g of iodine free salt was added and mixed well using a spatula. 2 capsules containing 15g of brine shrimp eggs were added to the tank and left undisturbed for 5 mins for proper soaking in salt water. After that airline tip was placed inside the artemia tank and the aeration level was increased to maximum level according to the manufacturers' instructions. After 24 hrs of incubation, the naupliis were hatched out from the brine shrimp eggs and observed using a stereomicroscope. Five tubes were taken and filled with 3ml of artificial sea water.10 naupliis were added in each test tube respectively. Test solutions were loaded in the concentration range of 10µl, 20µl, 30µl, 40µl and 50µl. A control tube was prepared by adding 3ml of artificial seawater, 10 naupliis. The tubes were kept for 24 hrs incubation. After incubation, the live and dead naupliis were counted and lethality was assessed.

### Antimicrobial activity analysis:

100 ml of Muller-Hinton agar was prepared, sterilized and poured onto the petriplates. The plates were allowed for solidification. After solidification plates was swabbed with four different oral pathogens namely Streptococcus mutans, Staphylococcus aureus, Pseudomonas sp, Enterococcus faecalis. All microorganisms were maintained at 4°C and were isolated from the patients. After swabbing on each plate four wells were formed using a T shaped well cutter. In the first three wells the test suspension was loaded in the concentration of 25µl, 50µl, 150µl respectively. In the fourth well a standard antibiotic in the concentration of 30 µl was loaded and the plates were incubated at 37°C for 24 hrs and zone of inhibition was measured after incubation. For Candida albicans, 20ml of Rose Bengal was prepared, sterilised and poured on to a petri plate and allowed for solidification. After solidification plates was swabbed with Candida albicans. After swabbing on each plate four wells were formed using a T shaped well cutter. In the first three wells the test suspension was loaded in the concentration of 25µl, 50µl, 150µl respectively. In the fourth well a standard antibiotic in the concentration of 30 µl was loaded. The plates were incubated at 37°C for 48 hrs and zone of inhibition was measured after incubation. The microbiological procedure was repeated three times for each microorganism.

## Results and Discussion:

**Figure 1:**
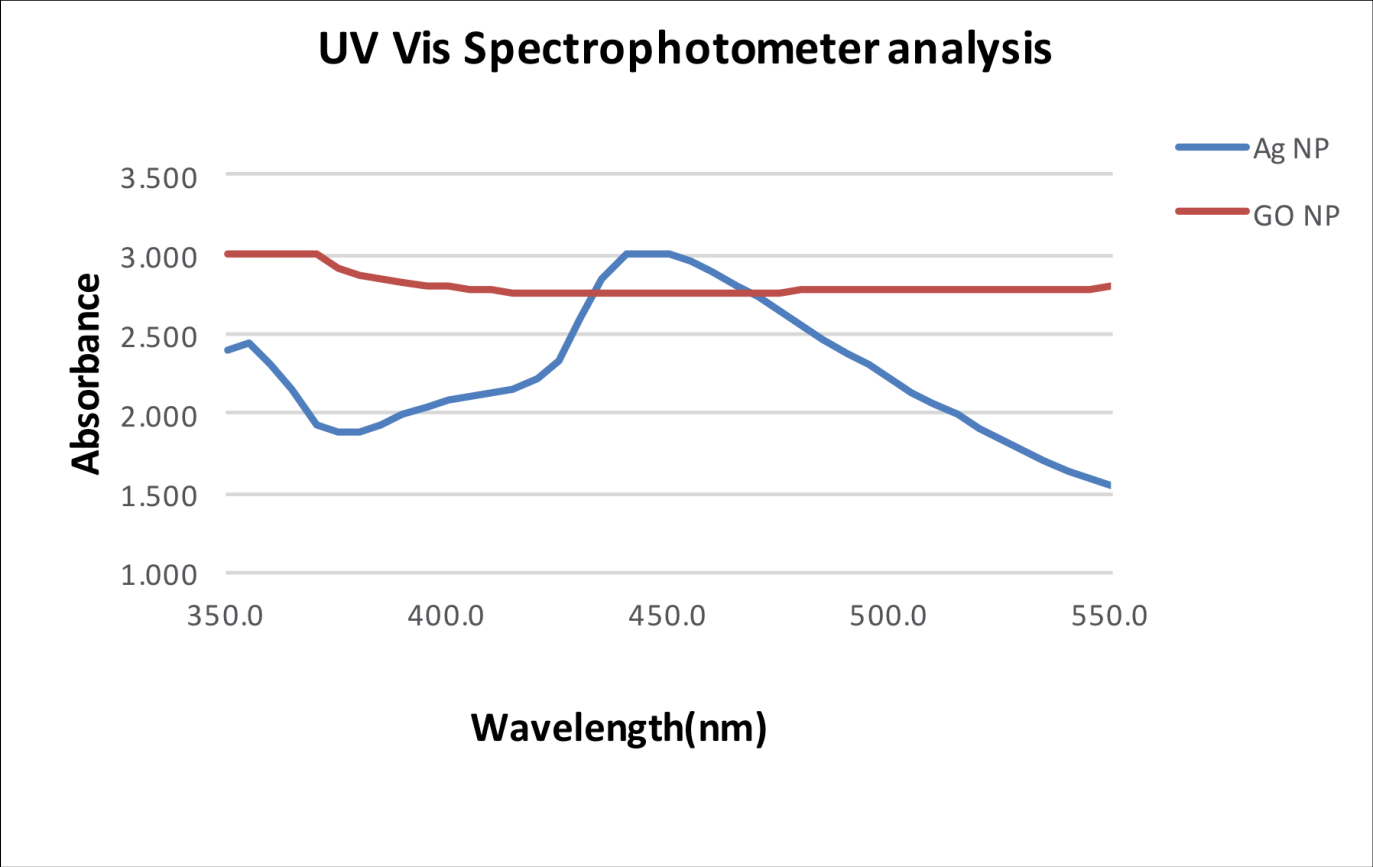
UV–Vis spectra of silver and graphene oxide nanoparticles.

**Figure 2:**
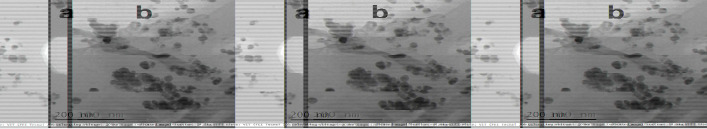
TEM images of (a) Silver nanoparticles and (b) Graphene Oxide nanoparticles

**Figure 3:**
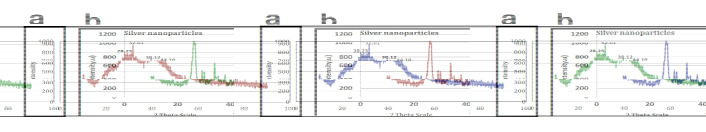
XRD patterns (a) Silver nanoparticles and (b) Graphene Oxide nanoparticles

**Figure 4:**
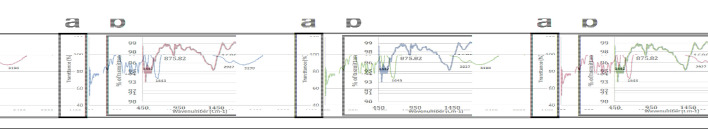
FTIR of (a) Silver nanoparticles and (b) Graphene Oxide nanoparticles

On UV Vis Spectrophotometer analysis the peak of 450 nm was observed which confirmed the synthesis of silver nanoparticles. The peak of 370 nm was observed for graphene oxide nanoparticles (Figure 1). TEM Analysis was done to confirm the shape and size of nanoparticles. Most of the silver nanoparticles were spherical in shape and average size of the silver nanoparticles was 11 to 55 nm (Figure 2). The graphene nanoparticles were spherical of 5-10 nm in size and rod shaped of 100 nm in size. On XRD analysis (Figure 3) the initial peaks were observed at 2θ values of 28.23° and 32.01°. These peaks are related to crystalline and amorphous organic phases of the plant extract. 2θ values of 38.12° and 44.10° was obtained which can be attributed to [111] and [200] crystalline planes of silver nanoparticles respectively and confirms the face centred cubic lattice of silver nanoparticles [8]. 2θ value was observed at around rGO (2θ=27°) corresponding to (002) plane [9]. FTIR analysis was done to check the possible functional groups present in the newly formed nanoparticles [10]. For silver nanoparticles the broad peak at 3190 cm-1 corresponds to O-H stretching vibrations of the hydroxyl groups. The peak at 2927 cm-1 is due to stretching vibrations of aliphatic C-H groups. The band at 1643 cm-1 corresponds to amide C=O stretching vibration. The peak at 1442 cm-1 attributes to H-C-H band for alkanes functional group. The observed peak at 1141 cm-1, 1288 cm-1 denote C-O stretching vibration. The peak at 1033 cm-1 can be attributed to C-O [carbonyl group] stretching vibration. FTIR analysis confirmed the formation of many oxygen-containing groups such as hydroxyl and carbonyl groups. From the results of FTIR it is evident that amide groups are formed along with carbonyl and hydroxyl group. It can be observed that silver has high affinity and binding ability with these carbonyl and hydroxyl groups and the interaction is taking place without any effect on the basic structure of silver during the course of the reaction. For graphene oxide nanoparticles the peak of 875.82 attributes to C-H bending and ring puckering bending vibration. The peak of 1408.35 attributes to O-H bending (in plane) vibration from hydroxyl groups. The absorption range of 1600.91 attributes to C=C (aromatic) stretching vibration which shows remaining sp2 character. The peak of 2272 attributes to C=N stretching vibration. The peak of 3028.34 attributes to C=C-H asymmetric stretching vibration (Figure 4). Brine shrimp lethality assay [11] was used to assess cytotoxicity. Silver nanoparticles were cytotoxic at all concentrations. As the concentration was increased the cytotoxic effects were more evident. The reduction in concentration may help to reduce cytotoxic effects. Graphene oxide showed no cytotoxicity at concentration of 10 µL, 20 µL. As the concentration was increased the cytotoxic effects were noted. Wierzbicki et al. assessed the cytotoxic effect of graphene oxide silver nanoparticle composite and found the combination seems to be less toxic when compared to silver nanoparticles used alone [12]. The biosynthesised silver nanoparticles were able to exert good antimicrobial activity against all the tested oral pathogens. For Streptococcus mutans at 50 µL the zone of inhibition was equivalent to the standard antibiotic. For Staphylococcus aureus and Pseudomonas sp at 25 µL itself zone of inhibition equivalent to the standard antibiotic was observed. At 150 µL Silver nanoparticles were able to achieve zone of inhibition equivalent to standard antibiotic for Enterococcus faecalis. For Candida albicans at all the concentrations zone of inhibition was not comparable to standard antibiotic. At all the concentrations of graphene oxide, there was zone of inhibition observed.8-10 mm zone of inhibition was observed at the concentration of 25 µL, 50 µL, and 150 µL. Antibiotic showed the maximum zone of inhibition of around 18 mm against tested microbes at all the concentrations. MA Aldosari et al synthesised Graphene silver nanoparticles nanocomposite by microwave irradiation method and tested their antimicrobial activity against E. coli and found microwave method to have better antimicrobial properties when compared to in situ-prepared nanocomposite [13]. The present study appear to be first of its kind as the combination of leaf extracts of Andrographis paniculata and Ocimum sanctum Linn to synthesise Silver and Graphene oxide nanoparticles have not been reported previously in the literature. Cobos et al synthesised graphene oxide decorated silver nanoparticles of size less than 4 nm using L ascorbic acid as reducing agent [14]. Silver nanoparticles exert its antimicrobial activity by creating pits in the cell wall of bacteria, which in turn increases permeability resulting in cell death. They also cause oxidation and denaturation of the cell wall of microorganisms, which results in cell lysis [15]. Graphene acts as a nano-knife, penetrates and cuts cell membrane of bacteria, induces mechanical stress, extracts phospholipids from lipid membranes and produces oxidative stress through ROS generation and by charge transfer phenomena [16].

## Conclusion

The silver and graphene oxide nanoparticles using leaf extract of Andrographis paniculata and Ocimum sanctum Linn. as reducing agents were synthesized for antimicrobial activity against common oral pathogens with acceptable cytotoxic effects. However, its effect on Candida albicans is insignificant. A combination of silver and graphene oxide nanoparticles analysis for antimicrobial activity is required for further validation and application.
